# Partitioning of parental histones between leading and lagging strands is modulated by DNA polymerase dosage

**DOI:** 10.1093/nar/gkag787

**Published:** 2026-08-12

**Authors:** Quinn Dickinson, Srinivasu Karri, Odile Djom Kenmogne, Shuang Zhu, Juan Carlos Rivera-Mulia, Zhenqing Ye, Zhiquan Wang, Haiyun Gan, Chuanhe Yu

**Affiliations:** Hormel Institute, University of Minnesota, Austin, MN 55912, United States; Hormel Institute, University of Minnesota, Austin, MN 55912, United States; Health Sciences Program, University of Minnesota, Rochester, MN 55901, United States; School of Life Sciences and Biopharmaceutics, Guangdong Provincial Key Laboratory of Pharmaceutical Bioactive Substances, Guangdong Pharmaceutical University, Guangzhou 510006, China; Department of Biochemistry, Molecular Biology and Biophysics, University of Minnesota Medical School, Minneapolis, MN 55455, United States; Lillehei Heart Institute, University of Minnesota Medical School, Minneapolis, MN 55455,United States; Division of Hematology, Department of Medicine, Mayo Clinic, Rochester, MN 55905, United States; CAS Key Laboratory of Quantitative Engineering Biology, Guangdong Provincial Key Laboratory of Synthetic Genomics and Shenzhen Key Laboratory of Synthetic Genomics, Shenzhen Institute of Synthetic Biology, Shenzhen Institutes of Advanced Technology, Chinese Academy of Sciences, Shenzhen 518055, China; Hormel Institute, University of Minnesota, Austin, MN 55912, United States

## Abstract

Epigenetic inheritance relies on the precise recycling of parental histones during DNA replication. The N-terminal region of Pol1, the catalytic subunit of DNA polymerase α, has previously been implicated in chaperoning parental histone recycling. In this study, we identify a single amino acid mutation in the C-terminal region of *Saccharomyces cerevisiae* Pol1 (*pol1-F1396S*) that destabilizes the protein and disrupts the transfer of parental histones to the lagging strand during DNA replication. As a primary consequence, *pol1-F1396S* cells exhibit decreased chromatin stability. Furthermore, we demonstrate that the cellular abundance—or dosage—of all three DNA polymerases (Pol α, Pol δ, and Pol ε) influences the distribution of parental histones between the leading and lagging strands, revealing a dosage-sensitive mechanism of epigenetic regulation during DNA replication.

## Graphical Abstract

**Graphical Abstract:**
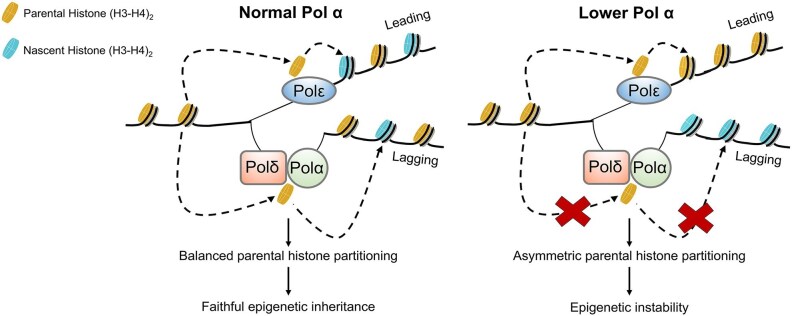

## Introduction

Faithful duplication of the eukaryotic genome is essential for genetic stability and proper cell function. Equally important is the preservation of the complex chromatin landscape, which regulates gene expression and underlies cell differentiation and development. The inheritance of both genetic and epigenetic information is tightly coupled to the DNA replication process [1–3]. Eukaryotic DNA replication is carried out by a multiprotein replisome, which unwinds parental DNA and synthesizes two daughter strands—the leading and lagging strands. The CMG helicase complex (Cdc45–MCM–GINS) unwinds the DNA template at replication forks [4]. DNA polymerase ε (Pol ε) synthesizes the leading strand, whereas DNA polymerase α (Pol α), together with a primase subunit, initiates RNA primers and Okazaki fragments that are subsequently extended by DNA polymerase δ (Pol δ) during lagging strand synthesis [1, 5–8]. The sliding clamp PCNA (Proliferating Cell Nuclear Antigen) enhances the processivity of both Pol ε and Pol δ and coordinates DNA synthesis with DNA repair and chromatin factors [9–12].

Eukaryotic chromosomes are packaged into nucleosomes, which consist of ∼146 bp of DNA wrapped around a histone octamer containing two copies each of H2A, H2B, H3, and H4. Replication of chromatin is thus inherently more complex than copying naked DNA [13]. During semiconservative DNA replication, approximately half of the histones assembled on newly synthesized DNA are newly synthesized, while the other half originate from parental histones “recycled” from ahead of the fork. Recycling of parental H3–H4 tetramers is particularly important because these histones carry epigenetic modifications that must be faithfully transmitted to daughter strands [14–16]. Notably, H3–H4 tetramers (referred to as parental histones) are thought to remain intact and are not split into dimers during their transfer to nascent DNA. H2A–H2B is thought to be released from chromatin as dimers [17], though this view has been challenged by recent observations of a histone hexamer at the replication fork [18].

Both biochemical and genetic studies have revealed that parental histone recycling is highly regulated during DNA replication. Distinct pathways mediate the partitioning of histones between leading and lagging strands. On the leading strand, the nonessential Pol ε subunits Dpb3 and Dpb4 act as histone chaperones that directly bind and transfer parental histones to the new DNA strands [19, 20]. On the lagging strand, parental histone recycling involves a sequential mechanism requiring Mcm2 (a histone-binding subunit of the MCM helicase), Pol1 (the catalytic subunit of Pol α), and Pol32 (a subunit of Pol δ) [21–26]. The replisome scaffold protein Ctf4 influences histone recycling efficiency on the lagging strand, likely by bridging the MCM helicase with Pol α [22]. Additional factors, such as the fork protection protein Mrc1 and the chromatin remodeler FACT (Facilitates Chromatin Transcription), contribute to parental histone transfer to both strands [18, 27–29].

Although defects in these pathways do not grossly impair DNA synthesis or cell proliferation [19, 24], they profoundly affect chromatin organization and cell physiology. Asymmetric partitioning of parental histones has been shown to (i) alter chromatin structure and gene expression patterns in yeast and other organisms [19, 24, 30, 31]; (ii) lead to the accumulation of free histones in yeast, which can inhibit homologous recombination [32]; (iii) impair embryonic stem cell differentiation and embryonic development in mice [20, 33]; and (iv) increase epigenetic and transcriptional heterogeneity in cancer cell lines [34]. These findings underscore the critical role of balanced parental histone recycling in maintaining chromatin stability and epigenetic inheritance [14–16, 35].

Despite identification of the core histone chaperones and pathways involved, the molecular factors that regulate the balance of parental histone partitioning between leading and lagging strands remain poorly understood. Here, we report the discovery of a point mutation in the catalytic subunit of DNA Pol α (*pol1-F1396S*) that reduces Pol1 levels but does not alter histone binding. The reduced Pol1 abundance shifts parental histone recycling toward the leading strand and results in decreased chromatin stability. Furthermore, experimentally altering the dosage of any one of the three major DNA polymerases [Pol1, Pol2 (the catalytic subunit of Pol ε) and Pol3 (the catalytic subunit of Pol δ)] directly influences the partitioning of parental histones during replication. These findings reveal a previously unrecognized dosage-sensitive regulatory mechanism that couples DNA replication dynamics with chromatin inheritance, providing new mechanistic insight into how a single genetic mutation can globally reshape epigenetic landscapes.

## Materials and methods

### Yeast strains, growth conditions, and construction

*Saccharomyces cerevisiae* yeast strains were grown in YPD (2% peptone, 1% yeast extract, 2% glucose) or yeast Synthetic Complete minus tryptophan (SC-Trp) media (for CRASH assay), at 30°C [36]. Yeast strains (W303 background) used in this study are listed in Supplementary Table S1.

Pol1 mutant strains were generated using a one-step polymerase chain reaction (PCR) procedure with Pol1-specific primers (Supplementary Table S2) [37, 38]. For degron strain construction, we utilized the HO locus targeting plasmid OsTIR1w/oGFP (Addgene# 102 883) [39]. The plasmids were linearized and transformed into wild–type (WT) strain cyc560 to generate the starter strain cyc970 [26], which was subsequently used to construct the degron strains via a PCR-based tagging method [38]. The standard lithium acetate transformation method was used for yeast transformation [40]. The addition of a 9Myc tag to POL1 and the insertion of the NatR marker did not compromise cell growth, with mutant strains displaying colony sizes comparable to the WT W303 control.

### Isolation of *pol1-C1372S,F1396S*

While investigating the Fe-S cluster-binding motif of Pol1 using a *pol1-C1372S* substitution [41], we identified a spontaneous second-site mutation, *pol1-F1396S*. Interestingly, the presence of this second mutation resulted in a dramatic enhancement of the histone transfer defects compared to the C1372S mutation alone. This prompted us to decouple the mutations and focus on *pol1-F1396S*, which we found to be a novel and essential factor in maintaining epigenetic stability through its role in Pol1 protein stability.

### Western blot

To monitor the levels of Pol1 protein level in yeast strains, we performed whole cell extraction using Tris–buffered saline (20 mM Tris pH 7.5, 150 mM NaCl) with protease inhibitor phenylmethylsulfonyl fluoride (PMSF). Briefly, log-phase cells (5 OD_600_) were harvested by spinning cultures at 5000 rpm (revolutions per minute), then washing the pellets with water, heating them at 95°C for 3 min, and suspending them in 50 ml tris-buffered saline buffer with 2 mM PMSF. Cells were physically broken by glass bead–beating them for two cycles (30 seconds per cycle). Proteins were then solubilized in 50 ml of 2× sodium dodecyl sulfate (SDS) sample buffer [Tris–Cl (pH 6.8), 4% (w/v) SDS (electrophoresis grade), 0.2% (w/v) bromophenol blue, 20% (v/v) glycerol]. Insoluble debris was separated from supernatant by spinning the samples at 15 000 × *g* speed. Soluble proteins in the supernatant were then separated by sodium dodecyl sulphate–polyacrylamide gel electrophoresis, transferred to nitrocellulose membrane (Bio-Rad, 1620 115) using Tris–glycine buffer, and probed with specific primary antibodies [anti-c-Myc antibody (9E10 M4439, Sigma), yeast PCNA (provided by Dr Zhiguo Zhang) [10], PGK1 (22C5D8 ab113687, Abcam)]. The secondary antibodies were either goat anti-rabbit IgG horseradish peroxidase (HRP; AB_2 337 913, Jackson Immunoresearch) or goat anti-mouse IgG HRP (AB_10 015 289, Jackson Immunoresearch). Immunoblots were developed by using Chemiluminescent Substrate (PI37069, Thermo Scientific) and images acquired using Bio-Rad Chemidoc (12 003 154, Bio-Rad). The sample collection and process with Rad53-P followed previous publications [32, 42]. Blotting was performed with anti-Rad53 antibody (ab104232, Abcam).

### Cycloheximide chase assay

For cycloheximide (CHX) chase experiments, yeast cells were cultured in YPD medium to mid-log phase. Equal cell numbers (OD₆₀₀ = 10) were resuspended in pre-warmed YPD containing CHX (Sigma–Aldrich, C7698) at a final concentration of 250 µg/ml and incubated for 0, 20, 40, or 60 min. At each time point, cells were harvested by centrifugation for western blotting.

### Cell cycle analysis with flow cytometry

Yeast strains were grown in YPD medium at 30°C until the cells reached mid-log phase (∼0.6 OD at 600 nm). Two doses of the mating pheromone α factor (5 µg/ml; EZBiolab) were added for 3 h at 25°C to arrest cell growth at the G1 phase. Cells were washed twice with cold water and then released into fresh YPD medium at 25°C. α factor was added 60 min after release from G1-phase (as Fig 2A) so that completion of cell division could be monitored by the disappearance of 2C cells and the reappearance of 1C cells. A total of ∼1 × 10^7^ cells were harvested at various time points by centrifugation, washed with water, and fixed with 70% ethanol overnight at 4°C. The sample treatment and flow cytometry were performed according to our previous publications [32, 43].

### Analysis of silencing-loss at the HoMothallism Left locus using the CRASH assay

The yeast strains were used to measure the apparent silencing-loss rate at the HoMothallism Left (HML) locus [30, 43, 44]. Briefly, 10 colonies of each strain were grown separately overnight in SC-Trp media, diluted to 0.01 OD_600_ in SC-Trp media, and grown for 5 h at 30°C. Cells were treated with 20 µM nicotinamide for the green fluorescent protein (GFP)+ control; cells were grown in hygromycin (200 µg/ml) for the red fluorescent protein (RFP)+ control. The apparent silencing-loss rate at the HML locus was calculated by dividing the number of RFP+ GFP+ cells (cells that have recently undergone Cre-mediated recombination express GFP but not RFP) by the total number of cells with the potential to lose silencing (RFP+ GFP− and RFP+ GFP+). For each colony, 50 000 events were analyzed using a BD Fortessa cytometer.

### Chromatin immunoprecipitation and enrichment and sequencing of protein-associated nascent

We performed histone enrichment and sequencing of protein-associated nascent (eSPAN) according to the protocol described in our previous studies [26, 45–47], with some modifications. For cell culture, yeast cells were grown in YPD- medium to exponential growth phase (OD_600_ ∼ 0.5). Two doses of α-factor (5 µg/ml; EZBiolab) were added over a 3-h incubation at 25°C to arrest cell growth at the G1 phase. Cells were washed twice with cold water and then released into fresh YPD medium containing 400 mg/l bromodeoxyuridine (BrdU), with or without 200 mM hydroxyurea. For the hydroxyurea-treated samples, cells were incubated for an additional 45 min. Hydroxyurea stalls the replication fork but does not interfere with assembly of newly synthesized and parental histones [19, 22, 26].

Cells were fixed with freshly prepared paraformaldehyde (final concentration: 1%) at 25°C for 20 min, followed by quenching with 0.125 M glycine for 5 min at room temperature. Fixed cells were collected by centrifugation at 3000 rpm for 5 min and washed twice with cold water. For chromatin immunoprecipitation (ChIP), cells were lysed in 0.1 ml ChIP lysis buffer [50 mM HEPES (pH 8.0), 150 mM NaCl, 2 mM ethylenediaminetetraacetic acid, 1% Triton X-100, 0.1% sodium deoxycholate] with glass beads using the Mini-Beadbeater-16 (Glen Mills Inc.). Broken cells were collected and resuspended in the ChIP lysis buffer. Next, lysates were sonicated for 15 cycles (Bioraptor Pico machine, 30 s ON/OFF) at 4°C to release chromatin fragments into solution. Soluble chromatin was immunoprecipitated with anti-H3K4me3 antibody (0.2 μg/ml; ab8580 Abcam), anti-H3K56Ac antibody [48] (0.05 μg/ml; from Dr Zhiguo Zhang’s lab) or RPA1 antibody (0.3 μg/ml; gift of Steven Brill). Immunocomplexes were recovered using prewashed Protein G Sepharose beads (17-0618-02, GE Healthcare). After extensive washing, ChIP DNA and cell lysis input was recovered by overnight reverse crosslinking. DNA was then purified and subjected to end-repair and adapter (IDT, Cat# 10 005 268) ligation using the KAPA hyper prep kit (Roche, Cat# 07 962 363 001). After saving 1% for ChIP-seq analysis, the remaining products were subjected to BrdU immunoprecipitation (BrdU-IP). Briefly, ligated DNA was denatured at 100°C for 5 min and then snap-cooled on ice for 5 min. Then DNA was diluted with 10 volumes of BrdU IP buffer [1× phosphate buffered saline, 0.0625% Triton X-100 (v/v)]. Anti-BrdU antibody (0.17 μg/ml; 555 627, BD Biosciences) was added, and samples were incubated at 4°C for 2 h. Next, 20 µl prewashed Protein G beads were added and incubated for an additional hour at 4°C. After extensively washing, bead-bonded DNA was eluted with 100 μl 1× TE buffer [10mM Tris-HCL (pH 8.0),1mM EDTA] containing 1% SDS and purified using a QIAGEN MinElute PCR Purification kit. The resulting eSPAN and BrdU-IP products, together with the input and ChIP-DNA, were amplified with the KAPA hyper prep kit and indexing primers [46].

### Sequence mapping and data analysis

Sequence mapping, nucleosome mapping, BrdU-IP-ssSeq (BrdU immunoprecipitation plus strand-specific next-generation DNA sequencing), RPA-ChIP-ssSeq (RPA chromatin immunoprecipitation plus strand-specific next-generation DNA sequencing) and eSPAN analysis were performed similarly to what has been previously described [19, 22, 49, 50]. Briefly, reads were trimmed with Cutadapt [51], merged with ngmerge [52], mapped to sacCer3 reference genome with Bowtie2 software [53] and forward (Watson strand) and reverse (Crick strand) alignments were separated with Samtools [54]. Using deeptools [55], coverage for these separate filtered reads was determined. To calculate the eSPAN bias pattern, the ratio of these reads was calculated around the DNA replication origins using pre-defined nucleosome positions [56]. Total eSPAN sequence reads at ±10 nucleosomes surrounding the DNA replication origins were counted using self-developed python scripts. The log_2_ ratio of Watson over Crick strand reads at each nucleosome position was used to obtain the average eSPAN bias pattern.

## Results

### *pol1-F1396S* mutant alters parental histone transfer patterns

**Figure 1.**
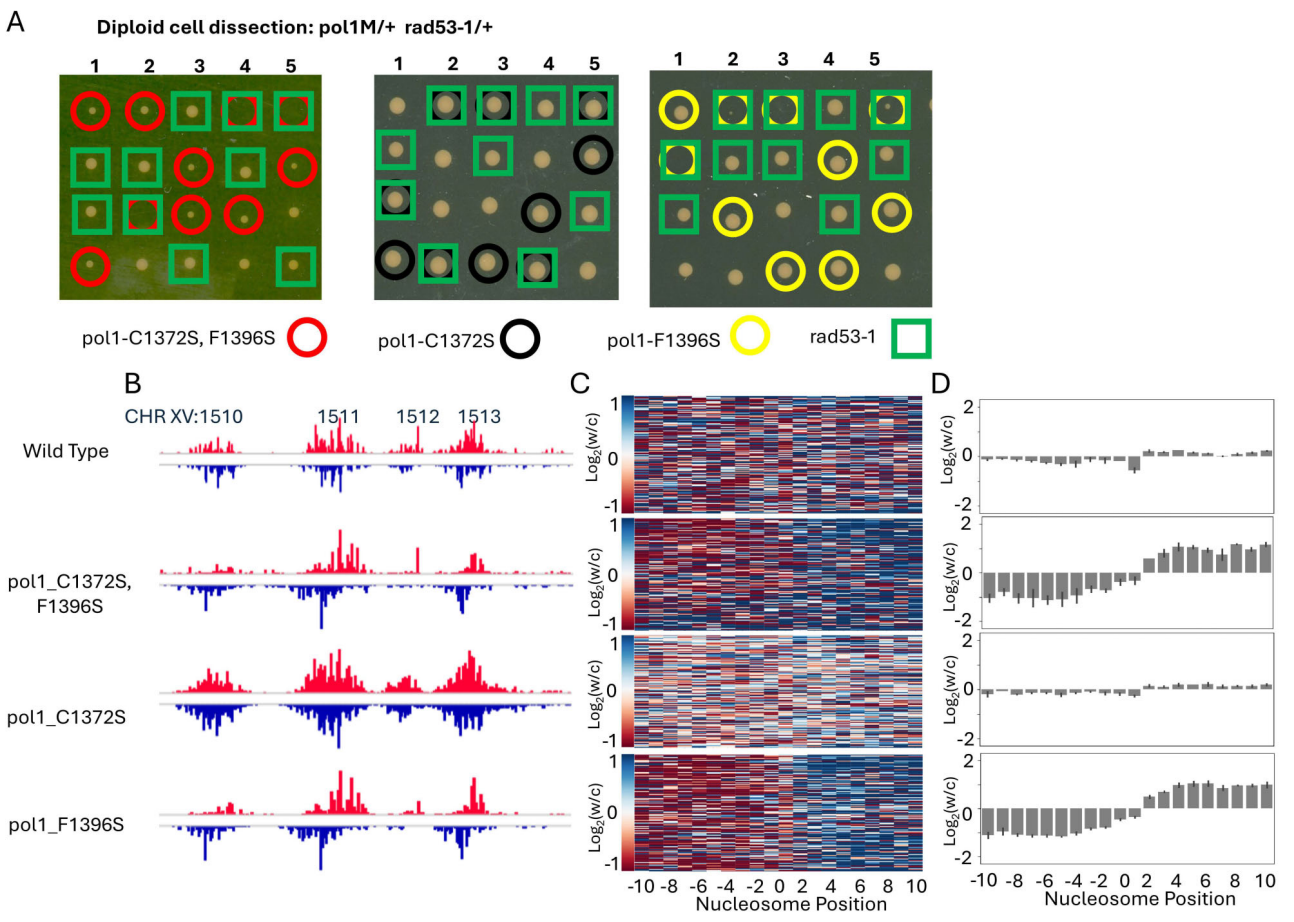
*pol1-F1396S* mutant alters parental histone transfer pattern. (**A**) Tetrad dissection of diploid strains (*pol1-M/+, rad53-1/+*). Left panel, *pol1-C1372S,F1396S*; middle panel, *pol1-C1372S*; right panel, *pol1-F1396S*. The *Pol1* genotype was inferred based on the linked nourseothricin (clonNAT) marker; *rad53-1* was identified by its hypersensitivity to hydroxyurea (data not shown) [59, 75]. For each dissection, five tetrads (1-5) are shown. All spores were grown on YPD for 2–3 days. (**B**) Snapshots of parental histone H3 (H3K4me3) eSPAN read enrichment at the replication origins ARS1510–1513 on chromosome XV in WT, *pol1-C1372S, pol1-F1396S*, and *pol1-C1372S,F1396S* strains. Watson strand reads are shown in red; Crick strand reads in blue. The *pol1-F1396S* mutant data was repeated with three independent strains. The others have been repeated once. (**C**) Heatmaps showing the log₂ (Watson/Crick) strand bias of H3K4me3 eSPAN signal at 20 individual nucleosomes (−10 to +10) flanking the top 50 strongest early replication origins of average bias [43, 45]. Each row represents one origin; nucleosome positions are labeled at the bottom. (**D**) Average strand bias [log₂ (Watson/Crick)] of H3K4me3 eSPAN signal across the same 20 nucleosomes (−10 to +10) surrounding the top 50 early replication origins in WT and mutant strains. Data are represented as mean ± 95% confidence interval from two independent experiments.

While investigating the role of DNA Pol α (Pol1) in parental histone recycling in *S. cerevisiae*, we isolated a mutant strain (*pol1-C1372S,F1396S*) that displayed a slower growth phenotype. Tetrad dissections confirmed that slow growth was linked to the POL1 locus (Fig. 1A, left panel). Sanger sequencing revealed two point mutations in this strain, resulting in amino acid substitutions C1372S and F1396S (Supplementary Fig. S1). Because these residues lie in the C-terminus of Pol1, outside the known histone H3–H4 binding domain located at the N terminus [24, 57], we initially expected that these mutations would not affect parental histone recycling.

To test this, we performed histone H3 eSPAN (enrichment and sequencing of protein–associated nascent DNA) to examine potential changes in parental histone transfer strand bias [19, 45, 46]. Using previous established histone marks, H3K4me3 for parental histones and H3K56ac for newly synthesized histones [19, 58], we compared strand–specific patterns between WT and *pol1-C1372S,F1396S* strains (Supplementary Fig. S2A). In the double *pol1-C1372S,F1396S* mutant, H3K4me3 eSPAN revealed a strong bias toward the leading strand, in contrast to the nearly symmetric distribution observed in WT cells (Fig. 1B–D). Reciprocally, H3K56ac eSPAN showed a lagging strand bias in *pol1-C1372S,F1396S*, whereas WT cells showed no clear strand bias (Supplementary Fig. S2B–D). To determine which point mutations (C1372S and F1396S) were responsible for this altered pattern, we generated single mutants carrying either *pol1-C1372S* or *pol1-F1396S* (Fig. 1A, middle and right panels). H3K4me3 eSPAN analysis demonstrated that only the *pol1-F1396S* mutant recapitulated the strand bias phenotype of *pol1-C1372S,F1396S*, while the *pol1-C1372S* mutant behaved similarly to WT (Fig. 1B–D). These results support that *pol1-F1396S* mutation affects the parental histone transfer on the replication fork. This conclusion is also supported by the H3K56ac eSPAN (Supplementary Fig. S2B–D). In total, these results indicate that the F1396S mutation of Pol1 is responsible for a substantially altered parental histone transfer pattern.

### Effect of the Pol1 mutant on cell cycle, checkpoint activation, and genome stability

We next characterized the cell cycle and genome stability phenotypes of *pol1-C1372S, pol1-F1396S*, and *pol1-C1372S,F1396S*. First, we used flow cytometry to assess the cell cycle state of unsynchronized, log-phase cultures. Flow cytometry analysis showed a higher proportion of cells in S phase in both *F1396S* and *C1372S,F1396S* mutants compared to WT, suggesting slower replication kinetics in these mutants (Supplementary Fig. S3A). To confirm this, we synchronized cells at G1 phase with alpha factor and monitored their replication progression. The double mutant displayed a pronounced delay in S-phase progression, and *pol1-F1396S* showed a moderate delay (Supplementary Fig. S3B), indicating defective DNA replication kinetics.

These S-phase delays likely activate the S-phase cell cycle checkpoint. Genetic interaction assays showed that single *pol1-F1396S* or double *pol1-C1372S,F1396S* mutants exhibited synthetic lethality or severe growth defects when combined with the checkpoint-defective *rad53-1* mutant (Fig. 1A), indicating that the checkpoint pathway is essential for their viability. To directly assess checkpoint activation, we quantified Rad53 phosphorylation, a marker of checkpoint activation [59], via immunoblotting. Rad53 phosphorylation was strongly induced in the *pol1-C1372S,F1396S* mutant and weakly induced in the *pol1-F1396S* mutant as compared to the WT and the *pol1-C1372S* mutant (Supplementary Fig. S3C). Further, this activation is specific to S-phase and more prominent in the *pol1-C1372S,F1396S* mutant than in WT (Supplementary Fig. S3D) [60].

**Figure 2.**
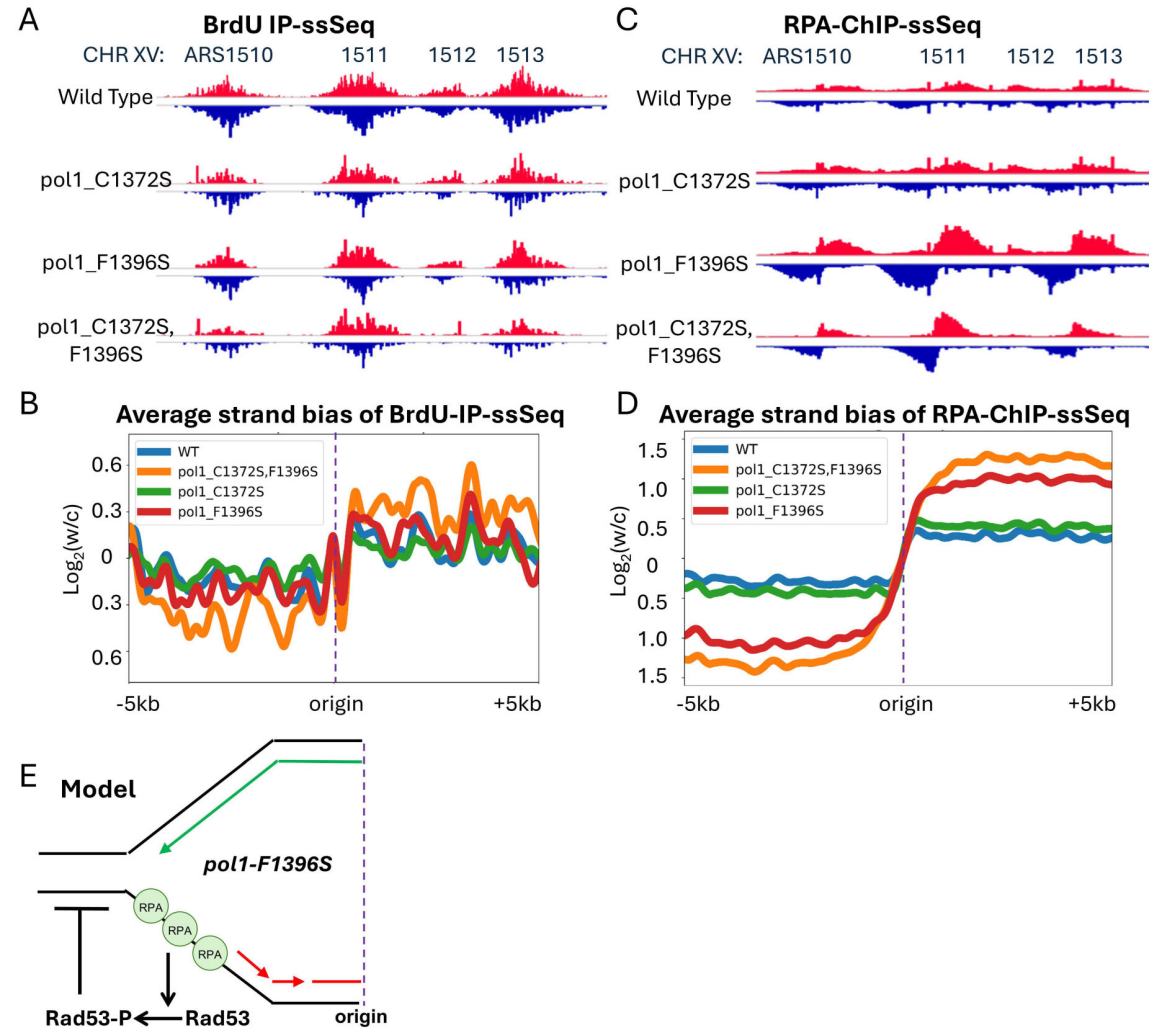
*pol1-F1396S* shows lagging strand gaps by BrdU-IP-ssSeq and RPA-ChIP-ssSeq analysis. (**A**) Snapshots of BrdU-IP-ssSeq reads at ARS1510–1513 in *WT, pol1-C1372S, pol1-F1396S*, and *pol1-C1372S,F1396S* strains. Watson strand reads are shown in red; Crick strand reads in blue. BrdU-IP-ssSeq monitor efficiency of the DNA synthesis. The *pol1-F1396S* mutant data was repeated with three independent strains. The others have been repeated once. (**B**) Average log_2_ (Watson/Crick) bias of BrdU-IP-ssSeq across the replication origins with a 200-bp scanning window. (**C**) Snapshots of RPA-ChIP-ssSeq reads at ARS1510–1513 in *WT* and *pol1-C1372S,F1396S* strains. RPA-ChIP-ssSeq was employed to monitor ssDNA accumulation. These experiments data was collected across two distinct time points for each run to ensure reproducibility. (**D**) Average log_2_ (Watson/Crick) bias of RPA-ChIP-ssSeq across the replication origins with a 200-bp scanning window. (**E**) Proposed model of replication fork structure in *pol1-F1396S*. In the *pol1-F1396S* mutant, impaired Pol1 enzymatic activity leads to persistent gaps on the lagging strand due to delayed Okazaki fragment synthesis. These single-stranded DNA regions are coated with RPA, triggering activation of the DNA damage checkpoint pathway. In response, the checkpoint pathway slows or pauses replication fork progression to maintain fork stability. However, in the absence of a functional checkpoint (e.g. *rad53-1* background), these unresolved gaps cause replication fork collapse and ultimately lead to cell death (Fig. 1A).

S phase checkpoint activation generally results from an extended long-stretch of single-stranded DNA (ssDNA) accumulation, coated with replication protein A (RPA) [61, 62]. To investigate this, we used BrdU-IP-ssSeq (BrdU immunoprecipitation plus strand-specific next-generation DNA sequencing) to analyze strand-specific DNA synthesis [45, 50]. The double mutant *pol1-C1372S,F1396S* and *pol1-F1396S* displayed a slightly stronger leading-strand bias than WT (Fig 2A and B), indicating impaired lagging strand synthesis. Additionally, RPA-IP-ssSeq (RPA immunoprecipitation plus strand-specific next-generation DNA sequencing), which marks exposed single-stranded DNA, exhibited even stronger leading-strand bias in the *pol1-C1372S,F1396S* and *pol1-F1396S* mutant (Fig 2C and D), suggesting that lagging strand synthesis defects lead to accumulation of ssDNA (Fig. 2E). These RPA-coated ssDNA regions likely trigger checkpoint activation to maintain replication fork stability (Fig. 2E).

We also assessed the genome stability of *pol1* mutants using DNA repair protein Rad52 foci, an indicator for DNA damage and the process of DNA repair [32, 63]. The *pol1-F1396S* mutant displayed a mild increase in Rad52 foci that was not statistically significant, while the *pol1-C1372S,F1396S* mutant exhibited significantly greater Rad52 foci compared to WT cells (Supplementary Fig. S4A and B), indicating elevated DNA damage in this mutant background.

### *pol1-F1396S* mutation leads to chromatin instability

**Figure 3.**
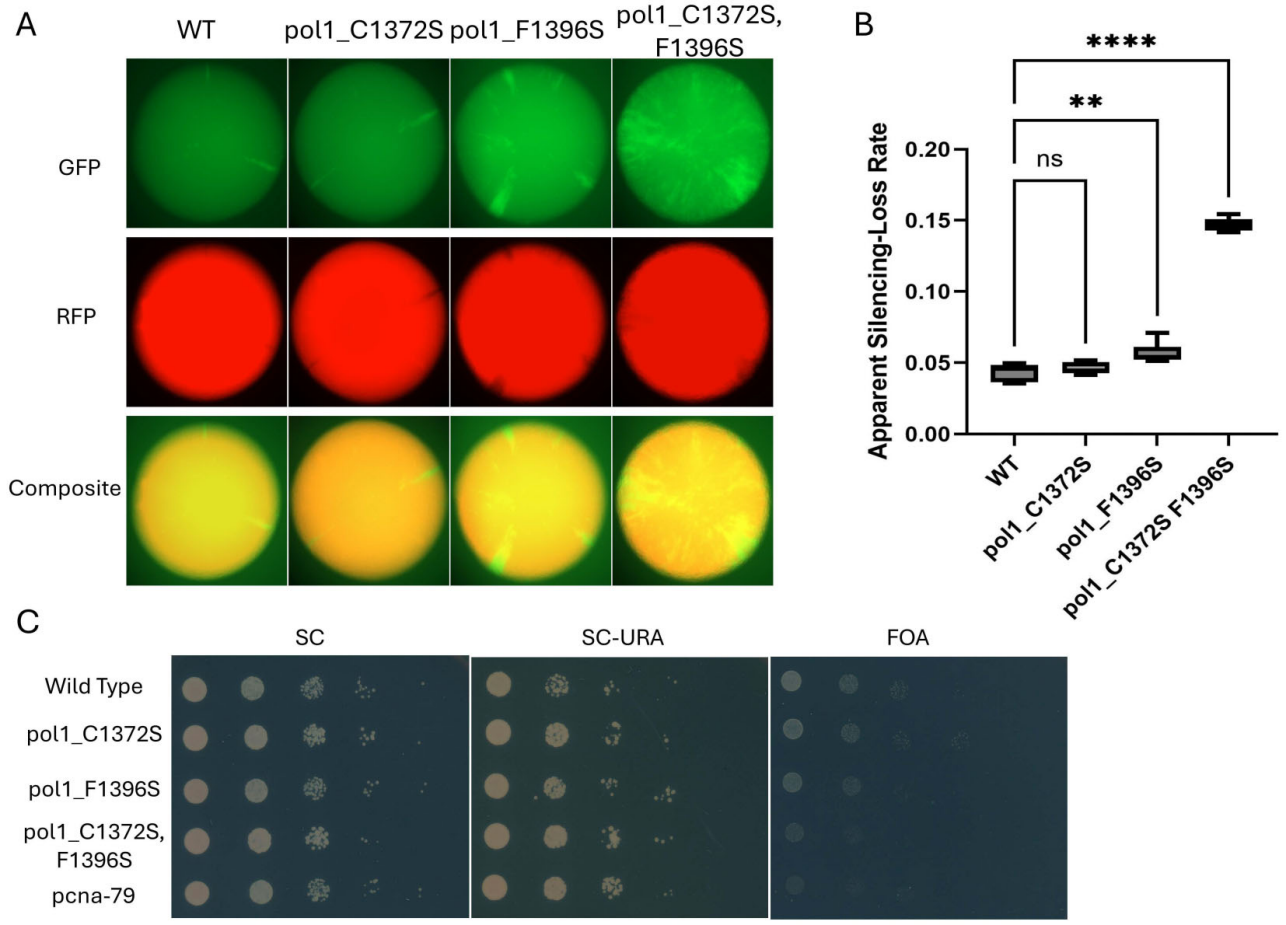
*pol1-F1396S* mutation leads to chromatin instability. (**A**) Representative fluorescence images of yeast colonies carrying the CRASH (Cre-reported altered states of heterochromatin) cassette (LoxP-RFP-LoxP-GFP at *URA3* and *HMLα::cre*) in WT, *pol1-C1372S, pol1-F1396S*, and *pol1-C1372S,F1396S* strains. Bright green sectors (GFP channel) or dark sectors (RFP channel) indicate loss of silencing at the HML locus. (**B**) Quantification of silencing defects illustrated in panel (A). Bars represent mean ± standard error. *P <.05, **P <.01, and ^****^P <.0001 by Student’s *t*-test. (**C**) The *pol1-F1396S* mutant reduced heterochromatin silencing at telomeric region. Ten-fold serial dilutions of the indicated strains were spotted on Synthetic Complete (SC), SC minus uracil (SC-URA), and SC + 0.1% FOA (fluoroorotic acid) plates. The *pol1-F1396S* and *pol1-C1372S,F1396S* alleles show decreased fitness on FOA, reflecting a failure to maintain heterochromatin at the telomere. These experiments were performed in duplicate to ensure reproducibility.

Defects in parental histone transfer generally lead to chromatin structure alteration and a change in gene expression patterns [16, 19, 64]. To evaluate the functional consequence of this histone transfer defect, we used the CRASH (Cre-Reported Altered States of Heterochromatin) system to monitor chromatin stability at the *HML* locus, which is maintained in a silent state through heterochromatin formation [30, 44]. Both *pol1-F1396S* and *pol1-C1372S,F1396S* strains showed increased sectoring, an indication of chromatin instability compared to WT, consistent with impaired histone transfer (Fig. 3A and B). The *pol1-C1372S* single mutant by contrast, behaved like WT. To determine if these stability defects extend to other heterochromatin regions, we examined the transcriptional silencing at telomeres using a URA3 reporter [65, 66]. In this assay, we used previous *pol30-79* mutant, a PCNA point mutation disrupts the silencing dependent on Asf1, as the positive control [10, 66]. We found that the *pol1-F1396S* and *pol1-C1372S,F1396S* mutants displayed increased sensitivity to 5-fluoroorotic acid (5-FOA) (Fig. 3C), indicating a failure to maintain heterochromatin. Interestingly, the *pol1-C1372S* mutant also showed increased sensitivity to 5-FOA at the telomeric regions. This suggests that the silencing defect in this specific mutant may arise through a mechanism independent of parental histone transfer. Collectively, these results demonstrate that the *pol1-F1396S* mutation disrupts chromatin stability across multiple genomic loci.

### *pol1-F1396S* functions in the same parental histone transfer pathway as *pol1-2A2*

**Figure 4.**
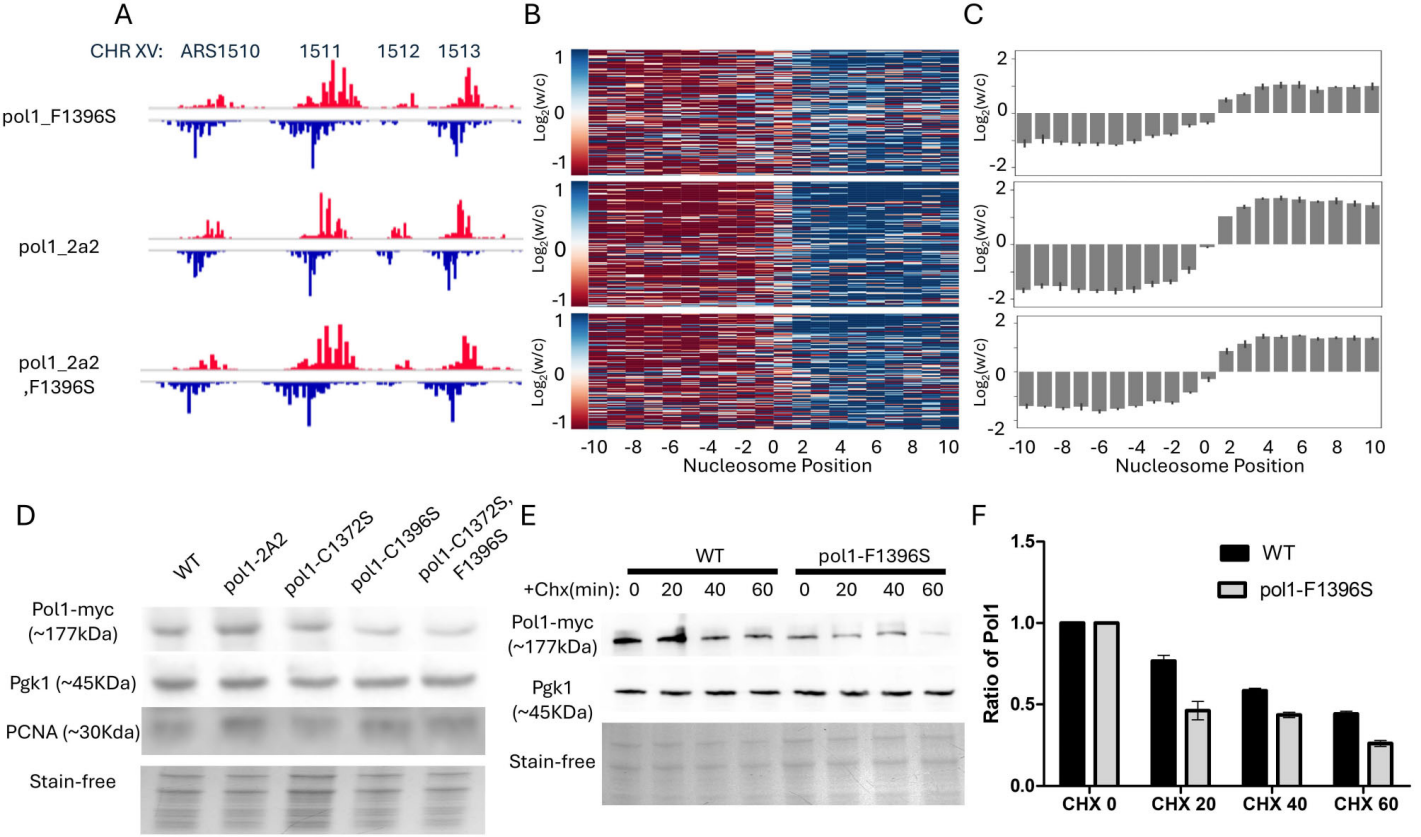
*pol1-F1396S* mutation functions in the same pathway as *pol1-2A2* on parental histone transfer and leads to Pol1 protein instability. (**A**) Snapshots of H3K4me3 eSPAN reads at replication origins ARS1510–1513 in *pol1-F1396S, pol1-2A2*, and *pol1-2A2,F1396S* strains. Watson strand reads are shown in red; Crick strand reads in blue. These experiments were performed in duplicate to ensure reproducibility. (**B**) Heatmaps showing strand bias of parental H3K4me3 at 20 nucleosomes flanking the top 50 early replication origins for each strain. The color codes the log2₂ (Watson/Crick reads number). (**C**) Average log_2_ (Watson/Crick) strand bias across the 10 nucleosomes in *pol1-F1396S, pol1-2A2*, and *pol1-2A2,F1396S* backgrounds. Data are represented as mean ± 95% confidence interval from two independent experiments. (**D**) Western blot showing Pol1 protein levels in WT, *pol1-C1372S, pol1-F1396S*, and *pol1-C1372S,F1396S* strains. Log-phase cultures were used. These Western experiments were performed in duplicate to ensure reproducibility. Molecular weight refers to Supplementary Fig. S5A. (**E**) CHX chase assay to monitor Pol1 degradation kinetics in WT and *pol1-F1396S*. Cells were treated with 250 µg/ml CHX for the indicated time points. Blots were probed with anti-Myc antibody (Pol1-Myc). Pgk1 and stain-free gel images were used as loading controls. Red boxes highlight the band for normalized Pol1 signal in panel (F). Molecular weight refers to Supplementary Fig. S5C. (**F**) Quantification of Pol1 signal decay from panel (E), normalized to loading controls (Pgk1). These western blot experiments were performed in triplicate to calculate the mean and standard deviation for the error bars.

Previous studies identified a histone-binding domain in the N-terminal region of Pol1, with the *pol1-2A2* mutation disrupting its interaction with histones and impairing parental histone recycling [24, 57]. Since *pol1-2A2* exhibited a similar leading strand bias to that in *pol1-F1396S* (Fig. 4A–C), we tested whether the two mutations act in the same pathway on the parental histone transfer. To do this, we constructed a double *pol1-F1396S-2A2* mutant and performed parental and new histone eSPAN analysis. If the mutations act in separate parallel pathways, we expect an additive effect on histone strand bias; if they act in the same pathway, the double mutant should resemble the single mutant with the stronger phenotype. Both H3K56ac and H3K4me3 eSPAN showed that the double mutant *pol1-F1396S-2A2* had a slightly reduced strand bias compared to *pol1-2A2* alone (Fig. 4A–C, and Supplementary Fig. S4C–E). These data suggest that *pol1-F1396S* acts within the same functional pathway as *pol1-2A2*, and its effect likely stems from impaired the histone chaperone function of Pol1.

### The *pol1-F1396S* mutation leads to Pol1 protein instability

Given that F1396 is situated away from the previously mapped histone-binding domain [24, 57], located within the C-terminus of Pol1, we sought to understand how this mutation disrupts parental histone transfer. Western blot analysis showed a marked reduction in Pol1 protein level in the *pol1-F1396S* mutant compared to both WT and the *pol1-C1372S* mutant (Fig. 4D and Supplementary Fig. S5A). The I-Mutant software predicted reduced stability (ΔΔG < 0) for both *pol1-F1396S* and *pol1-C1372S*, with a stronger effect for F1396S (Supplementary Fig. S5B) [67]. We then experimentally evaluated *pol1-F1396S* stability using a CHX chase assay. CHX treatment inhibits protein synthesis [68], and this assay measures a protein’s half-life. The Pol1-F1396S protein degraded more rapidly than WT Pol1 (Fig. 4E and F, and Supplementary Fig. S5C), confirming that the single *pol1-F1396S* mutation destabilizes the Pol1 protein.

### DNA polymerase dosage regulates parental histone transfer

**Figure 5.**
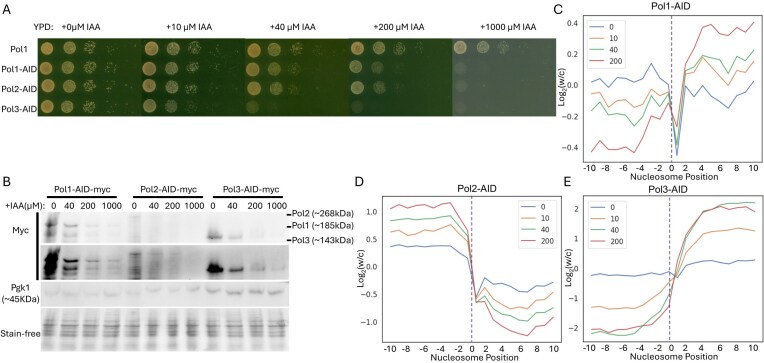
DNA polymerase dosage regulates parental histone transfer. (**A**) Growth assay of WT (cyc970) and AID-tagged strains [cyc1145 (Pol1-AID), cyc1147 (Pol2-AID), cyc1149 (Pol3-AID)] plated on YPD with varying IAA concentrations. (**B**) Western blot showing Pol1, Pol2, and Pol3 levels in AID-tagged degron strains treated with different concentrations of auxin (IAA) in YPD medium for 1 h. Molecular weight refers to Supplementary Fig. S5D. (**C**–**E**) Average strand bias (log_2_[Watson/Crick]) of H3K4me3 eSPAN signal at 10 nucleosomes surrounding the early replication origins in Pol1-AID (**C**), Pol2-AID (**D**), and Pol3-AID (**E**) strains under different IAA conditions (µM). Experimental workflow is shown in Supplementary Fig. S6A; genome browser snapshots and bias heatmaps are provided in Supplementary Fig. S6B–D. BrdU-IP-ssSeq is shown in Supplementary Fig. S7A and B.

To determine whether reduced expression of DNA polymerases in general affects parental histone transfer, we used a plant auxin-inducible degron (AID) system [38, 59] to titrate protein levels of Pol1 (Pol α), Pol2 (Pol ε), or Pol3 (Pol δ). Consistent with essential roles for Pol1–3 in DNA replication, all three degron strains exhibited growth defects at high indole-3-acetic acid (IAA) auxin concentrations (1000 µM IAA, Fig. 5A). The Pol3-degron strain showed the highest sensitivity, with growth defects even at low auxin levels (10 µM IAA). Western blotting confirmed that each polymerase could be efficiently degraded in an auxin concentration-dependent (Fig. 5B).

Next, we performed a parental histone eSPAN experiment to determine whether parental histone recycling is affected by the polymerase protein level. eSPAN samples were treated with 0–200 µM IAA for a half hour before release into early S phase (for details on sample collection, see Supplementary Fig. S6A). H3K4me3 eSPAN analysis showed that strains with reduced Pol1 and Pol3 protein levels exhibited a dose-dependent increase in the recycling of parental histones to the leading strand (Fig. 5C and E, and Supplementary Fig. S6B–D). Conversely, Pol2 depletion increased the recycling of parental histones to the lagging strand (Fig. 5D and Supplementary Fig. S6C). Interestingly, we also observed that AID-tagged Pol2 produced subtle changes in histone transfer bias even without auxin induction (0 µM IAA) (Fig. 5D and Supplementary Fig. S6C), suggesting that the presence of the AID tag alone may partially affect Pol ε function, possibly due to reduced basal expression or structural perturbation. These results collectively demonstrate that DNA polymerase dosage influences the partitioning of parental histones during replication, consistent with the established roles of Pol α, δ, and ε as histone chaperones.

In our parental histone eSPAN analysis with the DNA polymerases degron system, we also performed BrdU-IP-ssSeq to assess newly synthesized DNA strands. In Pol3-AID strains, BrdU-IP-ssSeq revealed a gradual increase in leading strand bias with rising auxin (IAA) concentrations, consistent with Pol3’s known role in lagging strand synthesis (Supplementary Fig. S7A and B). However, this trend was not observed in the Pol1-AID or Pol2-AID strains (Supplementary Fig. S7A and B). This discrepancy may reflect a greater sensitivity of Pol3 to IAA-induced depletion compared to Pol1 and Pol2. Notably, while BrdU strand bias remained largely unchanged in Pol1-AID and Pol2-AID cells (Supplementary Fig. S7A and B), parental histone eSPAN revealed significant strand bias alterations (Fig. 5C and E, and Supplementary Fig. S6B–D). This suggests that epigenetics is more sensitive to polymerase dosage than bulk DNA synthesis.

**Figure 6.**
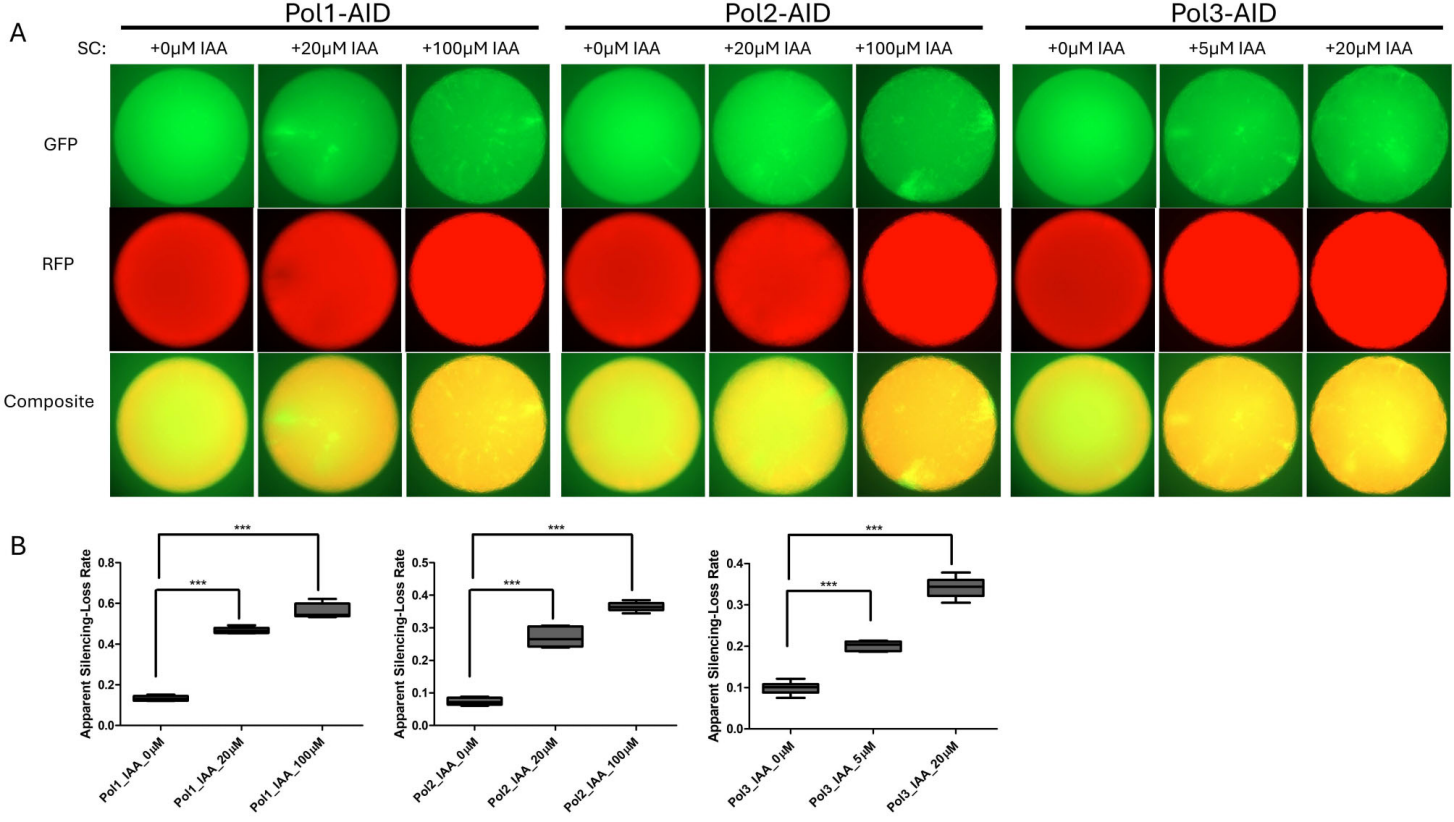
Reduced DNA polymerase dosage triggers chromatin instability. (**A**) Representative fluorescence images of yeast colonies carrying the CRASH (Cre-reported altered states of heterochromatin) cassette in Pol1, Pol2, and Pol3-AID-tagged degron strains [SK1066 (Pol1-AID), SK1068 (Pol2-AID), and SK10670 (Pol3-AID)] treated with different concentrations of auxin (IAA) in SC medium for 2 days. Bright green sectors (GFP channel) or dark sectors (RFP channel) indicate loss of silencing at the HML locus. These experiments were performed in duplicate to ensure reproducibility. (**B**) Quantification of silencing defects illustrated in panel (A). Bars represent mean ± standard error. *P <.05, **P <.01, and ^****^P <.0001 by Student’s *t*-test.

To determine whether reduced DNA polymerase levels compromise heterochromatin stability, we generated AID strains for each replicative polymerase (Supplementary Fig. S7C) and monitored the HML locus using the CRASH assay. Depletion of Pol1, Pol2, or Pol3 led to a significant increase in the frequency of silencing loss upon the addition of auxin (IAA) (Fig. 6A and B). These findings reinforce our conclusion that reduced DNA polymerase dosage triggers chromatin instability.

We also investigated the effect of polymerase titration on telomeric silencing. While we observed a clear reduction in growth on FOA plates following auxin induction, the interpretation was confounded by similar growth defects on the SC and SC-Ura control plates (Supplementary Fig. S8). DNA-polymerase degron strains exhibit a clear loss-of-silencing phenotype at the HML locus upon degradation, their effect at the telomeric region is less clear (Supplementary Fig. S8). Also, epigenetic silence pathways in *S. cerevisiae* operate via distinct mechanisms depending on the specific genomic locus [69]. This divergence between HML and telomere locus likely reflects a combination of locus-specific silencing dynamics and the severe confounding growth defects on plates resulting from the depletion of these essential replicative polymerases. Nevertheless, the CRASH assay results consistently demonstrate that a lower DNA polymerase dosage leads to a loss of heterochromatin stability at the HML locus.

## Discussion

In this study, we identified a point mutation in DNA Pol α (*pol1-F1396S*) that reduces Pol1 protein stability and expression and leads to altered parental histone H3–H4 recycling during DNA replication. Specifically, the mutation shifts parental histone partitioning from an unbiased state to a leading-strand bias. To further test the impact of replication factor dosage, we used an AID system to reduce the levels of Pol1, Pol2 (the catalytic subunit of Pol ε), and Pol3 (the catalytic subunit of Pol δ). In all three cases, we observed dose-dependent changes in the pattern of parental histone transfer. These results reveal that the abundance of DNA polymerases, whether altered by mutation or by induced degradation, can modulate the distribution of parental histones during replication and thereby influence chromatin structure.

Several replisome-associated proteins have previously been identified as key players in parental histone H3–H4 recycling [22, 25–29, 70]. These observations highlight that the replication fork is a functional hub that coordinates both DNA synthesis and histone inheritance. Our results show that even polymerases not directly known to bind parental histone H3–H4, such as Pol2 and Pol3, can affect parental histone partitioning when their protein expression is reduced. We propose that this effect is mediated through disruption of replisome assembly and function. For example, depletion of Pol2 may impair recruitment of the histone chaperone Dpb3/Dpb4 within Pol ε complex (Pol2–Dpb3/Dpb4), while reduced Pol3 could compromise the histone chaperone activity of Pol32 within the Pol δ complex (Pol3–Pol31–Pol32) [25, 26, 70]. Thus, the integrity of polymerase complexes is essential not only for DNA synthesis but also for maintaining balanced histone recycling. These findings underscore that epigenetic inheritance is sensitive to the structural dynamics of replication machinery. While all three replicative polymerases (Pol α, Pol δ, and Pol ε) are essential for DNA replication, they fulfill distinct roles at the fork: Pol α and Pol δ are primarily responsible for lagging-strand synthesis, whereas Pol ε is directly coupled with the CMG helicase to facilitate leading-strand progression. However, this division of labor is not absolute; evidence from catalytic-null *pol2* mutants suggests that Pol δ can support leading-strand synthesis in certain condition [71]. How such ‘polymerase switching’ or fork architectural changes influence the efficiency of parental histone transfer remains an open question. Future studies will be necessary to exploit these mutant backgrounds to further dissect the interplay between polymerase identity and epigenetic maintenance.

Our study also emphasizes the importance of protein abundance in regulating histone transfer. The *pol1-F1396S* mutation lies outside the known histone-binding domain [24, 57], yet it significantly reduces Pol1 protein levels and disrupts histone recycling. This suggests that epigenetic processes can be regulated by protein stability, independent of direct histone-binding mutations. More broadly, these observations imply that transcriptional regulation, messenger RNA stability, and translational control, any of which affect protein abundance, could influence chromatin inheritance mechanisms. This adds a new layer of regulatory complexity to how cells maintain epigenetic memory through cell division.

While mutations in DNA polymerases are typically associated with increased mutation rates and genomic instability [72], emerging evidence links such mutations to broader biological consequences [73]. Notably, a recent study identified mutations in human DNA Pol α (POLA1) as causative for X-linked intellectual disability (XLID) [74]. Several of these mutations were associated with reduced POLA1 protein levels [74]. These findings raise the possibility that defective epigenetic inheritance, rather than replication fidelity alone, may contribute to developmental disorders such as XLID.

Together, our results reveal that parental histone recycling is not only dependent on histone chaperone activity but also modulated by the expression and stability of DNA replication factors. These findings provide mechanistic insight into how chromatin structure can be reshaped by subtle genetic changes and raise the possibility that global epigenetic states may be modulated by replication-associated gene dosage effects. This expands our understanding of chromatin regulation and offers new perspectives for exploring how replication-linked defects may contribute to developmental abnormalities and diseases.

## Supplementary Material

gkag787_Supplemental_File

## Data Availability

The accession number for the sequencing data reported in this paper is GEO: GSE327335.
