# Supplementary material for: Partitioning of parental histones between leading and lagging strands is modulated by DNA polymerase dosage
Source: Nucleic Acids Res. 2026 Aug 12;54(15):gkag787. doi: 10.1093/nar/gkag787 (PMC13462271; doi:10.1093/nar/gkag787)
Supplement: Supplementary file 1 [file gkag787_supplemental_file.docx]

**Partitioning of Parental Histones Between Leading and Lagging Strands is Modulated by DNA Polymerase Dosage**

Quinn Dickinson^1†^, Srinivasu Karri^1†^, Odile Djom Kenmogne^2^, Shuang Zhu^3^, Juan Carlos Rivera-Mulia^4^, Zhenqing Ye^5^, Zhiquan Wang^6^, Haiyun Gan^7^, and Chuanhe Yu^1*^

^1^Hormel Institute, University of Minnesota, Austin, MN 55912 USA

^2^Health Sciences Program, University of Minnesota, Rochester, MN 55901 USA

^3^School of Life Sciences and Biopharmaceutics, Guangdong Provincial Key Laboratory of Pharmaceutical Bioactive Substances, Guangdong Pharmaceutical University, Guangzhou, 510006, China.

^4^Department of Biochemistry, Molecular Biology and Biophysics, University of Minnesota Medical School, Minneapolis, MN 55455 USA

^5^Lillehei Heart Institute, University of Minnesota Medical School, Minneapolis, MN 55455

^6^Division of Hematology, Department of Medicine, Mayo Clinic, Rochester, MN, 55905 USA

^7^CAS Key Laboratory of Quantitative Engineering Biology, Guangdong Provincial Key Laboratory of Synthetic Genomics and Shenzhen Key Laboratory of Synthetic Genomics, Shenzhen Institute of Synthetic Biology, Shenzhen Institutes of Advanced Technology, Chinese Academy of Sciences, Shenzhen, 518055, China

^†^The first two authors should be regarded as Joint First Authors

*Corresponding author:

Dr. Chuanhe Yu

**E-mail:** [yu000479@umn.edu](mailto:yu000479@umn.edu)

**Keywords:** DNA replication, DNA polymerase α, Parental histone recycling, Leading/lagging strand asymmetry, Epigenetic inheritance

Running title: DNA polymerase dosage regulates parental histone transfer

Supplementary Figures

**Supplementary Figure 1. Sanger sequence confirming the Pol1 mutants.**

**Supplementary Figure 2. H3K56Ac eSPAN in WT, *pol1-C1372S, pol1-F1396S*, and *pol1-C1372S,F1396S* strains**

**(A)** Schematic of sample collection and experimental workflow for histone eSPAN.

**(B)** Snapshots of new histone H3 (H3K56Ac) eSPAN signal at ARS1510-1513 in WT, *pol1-C1372S*, *pol1-F1396S*, and *pol1-C1372S,F1396S* strains. Watson strand reads are shown in red; Crick strand reads in blue. The *pol1-F1396S* mutant data was repeated with three independent strains
**(C)** Heatmaps showing log_2_(Watson/Crick) strand bias of H3K56Ac eSPAN signal at 20 nucleosomes (−10 to +10) flanking the top 50 early replication origins. Each row represents one origin.
**(D)** Average strand bias of H3K56Ac signal across the same nucleosome window in WT and mutant strains. Data are represented as the mean ± 95% confidence interval from two independent experiments.

**Supplementary Figure 3. Fluorescence-activated cell sorting (FACS) analysis of DNA content of *pol1* mutants and *pol1-C1372S,F1396S* mutant exhibits elevated checkpoint activation and DNA lesions during S phase**

**(A)** Cell cycle population analysis of *pol1* mutant. Flow cytometry results (left) and quantification (right) of cells in G1, S, and G2/M phases. Samples were collected from log-phase cell cultures.

**(B)** Cell-cycle progression analysis of WT, *pol1-C1372S*, *pol1-F1396S*, and *pol1-C1372S,F1396S* strains. Cell-cycle progression was monitored by flow cytometry after cells were released from arrest in G1-phase. A dose of mating pheromone was added 60 min after release from G1-phase so that completion of cell division could be monitored by the disappearance of 2C cells and the reappearance of 1C cells.

**(C)** Immunoblot showing increased Rad53 phosphorylation (Rad53-P) in *pol1-C1372S,F1396S*, but not in *pol1-C1372S*. A slight increase in Rad53-P is observed in *pol1-F1396S*. The samples were collected with the log-phase cell culture.
**(D)** Elevated Rad53-P levels in *pol1-C1372S,F1396S* during S phase cell release cells were released from arrest in G1-phase. Sample treatment and collection match the B panel.

**Supplementary Figure 4. Genome instability analysis of Pol1 mutant strains by Rad52 foci formation and *Pol1-F1396S* functions in the same pathway as *pol1-2A2*, based on H3K56Ac eSPAN analysis**
**(A)** Representative fluorescence images of Rad52-YFP foci (white arrows), indicating sites of DNA damage, in WT and mutant strains.
**(B)** Quantification of Rad52-YFP foci across >500 cells per strain. Statistical analysis was performed using one-way ANOVA. Asterisks indicate statistical significance between two strains. ns, P > 0.05; **,P < 0.01; ***, P  < 0.001; ****, P  < 0.0001. Error bars depict standard error of the mean.

**(C)** Snapshots of H3K56Ac eSPAN reads at ARS1510–1513 in *pol1-2A2*, *pol1-F1396S*, and *pol1-2A2,F1396S* strains. These experiments were performed in duplicate to ensure reproducibility.
**(D)** Heatmaps showing strand bias of H3K56Ac signal at 20 nucleosomes (−10 to +10) flanking the top 50 early replication origins.
**(E)** Average log₂(Watson/Crick) bias across the same nucleosome window as (D). Data are represented as the mean ± 95% confidence interval from two independent experiments.

**Supplementary Figure 5. *pol1-F1396S* mutation leads to Pol1 protein instability.**

**(A)** Western blot showing Pol1 protein levels in WT, *pol1-C1372S*, *pol1-F1396S*, and *pol1-C1372S,F1396S* strains. Referred to **Fig. 4D**.

**(B)** I-Mutant software predicts a reduction in stability for the *pol1-F1396S* protein compared to wild-type (WT).

**(C)** Cycloheximide (CHX) chase assay monitoring Pol1 degradation kinetics in WT and *pol1-F1396S* strains. Detailed quantification of protein half-life is shown in **Fig. 4E**.

**(D)** Western blot showing Pol1, Pol2, and Pol3 levels in AID-tagged degron strains treated with different concentrations of auxin (IAA) in YPD medium for one hour. Referred to **Fig. 5B**

**Supplementary Figure 6. H3K4me3 eSPAN analysis shows that DNA polymerase dosage modulates parental histone recycling**

**(A)** Schematic of the sample treatment workflow using the auxin-inducible degron (AID) system. Yeast cells were first arrested in G1 with α-factor for 2.5 hours, and treated with 0, 10, 40, or 200 µM IAA for 30 minutes, and then released into early S phase in YPD medium with 200 mM hydroxyurea, BrdU and accordingly IAA (0,10, 40, and 200 µM IAA) at 30°C for 45 minutes.

**(B-D)** Snapshots (upper panel) of H3K4me3 eSPAN read enrichment at ARS1510–ARS1513 in Pol1-AID(B), Pol2-AID(C), and Pol3-AID(D) strains under four auxin concentrations (0, 10, 40, and 200 µM IAA). Watson and Crick strand reads are shown in red and blue, respectively. Heatmaps (lower panel) showing log2(Watson/Crick) strand bias of H3K4me3 eSPAN at 10 nucleosomes flanking the top 50 early replication origins.

**Supplementary Figure 7. BrdU-IP-ssSeq analysis shows that Pol3 dosage modulates lagging strand DNA synthesis**

(A) Snapshots of BrdU-IP-ssSeq at ARS1510–1513 in Pol1-AID, Pol2-AID, and Pol3-AID strains under four auxin concentrations (0, 10, 40, and 200 µM IAA). The signals represent normalized sequence read density. The red and blue represent sequence reads of Watson and Crick strands, respectively. The strains used are the same as those in **Fig. 5**.

(B) Average strand bias of BrdU-IP-ssSeq peaks nearby replication origins in Pol1-AID, Pol2-AID, and Pol3-AID strains under four auxin concentrations (0, 10, 40, and 200 µM IAA). The average log_2_ ratios of sequence reads from Watson strand/Crick strand surrounding all early replication origins were calculated using a 200-bp sliding window. The strains used are the same as those in **Fig. 5**.

(C) Sensitivity of DNA polymerase-AID strains to auxin. Ten-fold serial dilutions of the control strain (SK1064) and the AID-tagged strains POL1-AID (SK1066), POL2-AID (SK1068), and POL3-AID (SK10670) were spotted onto YPD medium containing the indicated concentrations of auxin (IAA). These strains were utilized for the CRASH assays in panels (**Fig. 6A-B**) to evaluate the impact of reduced polymerase dosage on chromatin stability.

**Supplementary Figure 8. Analysis of telomeric silencing in DNA polymerase-AID strains.**

Ten-fold serial dilutions of WT (cyc1185), POL1-AID (cyc1588), POL2-AID (cyc1586), and POL3-AID (cyc1590) strains were spotted onto SC, SC-URA, and SC + 0.1% FOA media. While growth was reduced on FOA plates under auxin-induced conditions, similar growth inhibition on SC and SC-URA control plates suggests that the phenotype may be primarily driven by the essential requirement for DNA polymerases rather than a specific loss of telomeric silencing. These experiments were performed in duplicate to ensure reproducibility.

Tables

Table S1. Yeast strains used in this study

| **Strain** | **Genotype** | **References** |
| --- | --- | --- |
| cyc560 | *MATA ade2-1 ura3-1 his3-11,15 trp1-1 leu2-3,112 can1-100 BAR1(wt) + URA3::BrdU–Inc* | (1,2) |
| cyc999 | *MATA ade2-1 ura3-1 his3-11,15 trp1-1 leu2-3,112 can1-100 BAR1(wt) pol1_C1372S,F1396S::NatR+ URA3::BrdU–Inc* | this study |
| cyc1000 | *MATA ade2-1 ura3-1 his3-11,15 trp1-1 leu2-3,112 can1-100 BAR1(wt) pol1_C1372S::NatR+ URA3::BrdU–Inc* | this study |
| cyc1298 | *MATA ade2-1 ura3-1 his3-11,15 trp1-1 leu2-3,112 can1-100 BAR1(wt) pol1-2a2 + URA3::BrdU–Inc* | this study |
| cyc1532 | *MATA ade2-1 ura3-1 his3-11,15 trp1-1 leu2-3,112 can1-100 BAR1(wt) pol1_F1396S::NatR+ URA3::BrdU–Inc* | this study |
| cyc1534 | *MATA ade2-1 ura3-1 his3-11,15 trp1-1 leu2-3,112 can1-100 BAR1(wt) pol1_2A2_F1396S::NatR+ URA3::BrdU–Inc* | this study |
| cyc950 | *MATB ADE2 ura3-1 his3-11,15 trp1-1 leu2-3,112 can1-100 Rad52-YFP+ URA3::BrdU–Inc* | this study |
| cyc1564 | *ADE2 ura3-1 his3-11,15 trp1-1 leu2-3,112 can1-100 pol1_C1372S::NatR Rad52-YFP+ URA3::BrdU–Inc* | this study |
| cyc1244 | *MATB ade2-1 ura3-1 his3-11,15 trp1-1 leu2-3,112 can1-100 BAR1(wt) pol1_CF1396S::NatR Rad52-YFP+ URA3::BrdU–Inc* | this study |
| cyc1242 | *MATB ade2-1 ura3-1 his3-11,15 trp1-1 leu2-3,112 can1-100 BAR1(wt) pol1_C1372S,F1396S::NatR Rad52-YFP+ URA3::BrdU–Inc* | this study |
| JRY10790 | *MatA lys2 his3-11,15 leu2-3,112 can1-100 hml2alpha::cre ura3Δ::GPDpro-loxP-yEmRFP-CYC1term-hphMX-loxP-yEGFP-ADH1term* | (3) |
| cyc994 | *MatA lys2 his3-11,15 leu2-3,112 can1-100 hml2alpha::cre ura3Δ::GPDpro-loxP-yEmRFP-CYC1term-hphMX-loxP-yEGFP-ADH1term pol1_C1372S::NatR* | this study |
| cyc1566 | *lys2 his3-11,15 leu2-3,112 can1-100 hml2alpha::cre ura3Δ::GPDpro-loxP-yEmRFP-CYC1term-hphMX-loxP-yEGFP-ADH1term pol1_F1396S::NatR* | this study |
| cyc1568 | *lys2 his3-11,15 leu2-3,112 can1-100 hml2alpha::cre ura3Δ::GPDpro-loxP-yEmRFP-CYC1term-hphMX-loxP-yEGFP-ADH1term pol1_C1372S,F1396S::NatR* | this study |
| cyc1145 | *MATA ade2-1 ura3-1 his3-11,15 trp1-1 leu2-3,112 can1-100 BAR1(wt) Pol1-AIDMyc::HYG HO::ADH1p-OsTIR1-KanMX4 + URA3::BrdU–Inc* | this study |
| cyc1147 | *MATA ade2-1 ura3-1 his3-11,15 trp1-1 leu2-3,112 can1-100 BAR1(wt) Pol2-AIDMyc::HYG HO::ADH1p-OsTIR1-KanMX4 + URA3::BrdU–Inc* | this study |
| cyc1149 | *MATA ade2-1 ura3-1 his3-11,15 trp1-1 leu2-3,112 can1-100 BAR1(wt) Pol3-AIDMyc::HYG HO::ADH1p-OsTIR1-KanMX4 + URA3::BrdU–Inc* | this study |
| cyc970 | *MatA ade2-1 ura3-1 his3-11,15 trp1-1 leu2-3,112 can1-100 BAR1(wt) + URA3::BrdU–Inc HO::ADH1p-OsTIR1-KanMX4* | (4) |
| cyc1175 | *MATA ade2-1 ura3-1 his3-11,15 trp1-1 leu2-3,112 can1-100 BAR1(wt) pol1-F1396S-9Myc::Trp+ URA3::BrdU–Inc* | this study |
| cyc1526 | *MATA ade2-1 ura3-1 his3-11,15 trp1-1 leu2-3,112 can1-100 BAR1(wt) pol1-2a2-F1396S-9Myc::Trp + URA3::BrdU–Inc* | this study |
| cyc1528 | *MATA ade2-1 ura3-1 his3-11,15 trp1-1 leu2-3,112 can1-100 BAR1(wt) pol1-2a2-9Myc::Trp + URA3::BrdU–Inc* | this study |
| cyc1535 | *MATA ade2-1 ura3-1 his3-11,15 trp1-1 leu2-3,112 can1-100 BAR1(wt) Pol1-9Myc::Trp+ URA3::BrdU–Inc* | this study |
| cyc1574 | *MATA ade2-1 ura3-1 his3-11,15 trp1-1 leu2-3,112 can1-100 BAR1(wt) pol1_C1372S-9Myc::Trp+ URA3::BrdU–Inc* | this study |
| zgy323 | *pol30-79:TRP1, w303, URA3-VIIL* | (5) |
| cyc1185 | *MATA ade2-1 ura3-1 his3-11,15 trp1-1 leu2-3,112 can1-100 URA3-VIIL* | this study |
| cyc1579 | *MatA ade2-1 lys2 his3-11,15 leu2-3,112 can1-100 ura3-1 hml2alpha::cre pol1_C1372S::NatR URA3-VIIL* | this study |
| cyc1580 | *MatB ade2-1 lys2 his3-11,15 leu2-3,112 can1-100 ura3-1 hml2alpha::cre pol1_F1396S::NatR URA3-VIIL* | this study |
| cyc1583 | *MatA ade2-1 lys2 his3-11,15 leu2-3,112 can1-100 ura3-1 hml2alpha::cre? pol1_C1372S,F1396S::NatR URA3-VIIL* | this study |
| SK1064 | *MatA lys2 his3-11,15 leu2-3,112 can1-100 hml2alpha::cre ura3Δ::GPDpro-loxP-yEmRFP-CYC1term-hphMX-loxP-yEGFP-ADH1term his3-11,15 HO::ADH1p-OsTIR1-KanMX4* | this study |
| SK1066 | *MatA lys2 his3-11,15 leu2-3,112 can1-100 hml2alpha::cre ura3Δ::GPDpro-loxP-yEmRFP-CYC1term-hphMX-loxP-yEGFP-ADH1term his3-11,15 HO::ADH1p-OsTIR1-KanMX4 Pol1-AIDMyc::NatR* | this study |
| SK1068 | *MatA lys2 his3-11,15 leu2-3,112 can1-100 hml2alpha::cre ura3Δ::GPDpro-loxP-yEmRFP-CYC1term-hphMX-loxP-yEGFP-ADH1term his3-11,15 HO::ADH1p-OsTIR1-KanMX4 Pol2-AIDMyc::NatR* | this study |
| SK1070 | *MatA lys2 his3-11,15 leu2-3,112 can1-100 hml2alpha::cre ura3Δ::GPDpro-loxP-yEmRFP-CYC1term-hphMX-loxP-yEGFP-ADH1term his3-11,15 HO::ADH1p-OsTIR1-KanMX4 Pol3-AIDMyc::NatR* | this study |
| cyc1586 | *MatB ade2-1 lys2 his3-11,15 leu2-3,112 can1-100 ura3-1 Pol2-AIDMyc::HYG HO::ADH1p-OsTIR1-KanMX4 URA3-VIIL* | this study |
| cyc1588 | *MatA ade2-1 lys2 his3-11,15 leu2-3,112 can1-100 ura3-1 Pol1-AIDMyc::HYG HO::ADH1p-OsTIR1-KanMX4 URA3-VIIL* | this study |
| cyc1590 | *MatA ade2-1 lys2 his3-11,15 leu2-3,112 can1-100 ura3-1 Pol3-AIDMyc::HYG HO::ADH1p-OsTIR1-KanMX4 URA3-VIIL* | this study |

Table S2. Oligoes and primers used in this study.

| **Oligo** | **Oligo Sequence** |
| --- | --- |
| Pol1_C1372Sf | GTGTGATGACAGCACATGCGGTATAGTTACAAGACAAGTCTCCGTTTTTGGTAAGCGTTGTTTGAATGACGGCTCA ACGGGTGTCATGAGATACAAATACAGTGACAAGCAATTGTACAA |
| Pol1_C1372Sr | GTCTGTACATTAGGTATCTAGTAGGATGCGTATGTACAGTTGTATTC |
| Pol1natf | GAATACAACTGTACATACGCATCCTACTAGATACCTAATGTACAGAC CGG ATC CCC GGG TTA ATT AA |
| Pol1natr | TGATGCATGTGAGAACTGATCACCAGAGCCGAAAAAACATTCTCAGAATATCGCTGAAAACCTTCAATAATAAAAGCTTCCTAGCGCTCCTTCCGAT GAATTCGAGCTCGTTTAAAC |
| Pol1_2ndf | GGGCACACATTTCCTTATATTACGCAGGGTGGTTACAGTGTGATGACAGCACATGCGG |
| Pol1_2ndr | TCTTAATGCATGGGTTTATTAAAGGATGATGCATGTGAGAACTGATCACCAGAGC |
| PCR_Pol1r | GGGGTAGTCGAGATCATCGGGTAGATATATTGG |
| Pol1degronf | TCAAAAATATTTGAACGATTGTGGACGTCGCTACGTTGATATGACTAGCATATTTGATTTCATGCTAAAT CGTACGCTGCAGGTCGAC |
| Pol1degronr | ACAGTTGTATTCTTTTCTTACTATATAGAATATTCATGAGATCACACAACACATACAAAATACTTACCTA ATCGATGAATTCGAGCTCG |
| Pol2degronf | TGTGTTTAAGCAAGTAGCCAAGTATTACGGTTTTGATATATTATTGAGTTGTATTGCTGATTTGACCATA CGTACGCTGCAGGTCGAC |
| Pol2degronr | TTTTTTTTTTTTTTTTTTTTTTTTCATGGTAAAGAGGCCATTGAACCTCGCGTTATATACTGCTTACTCA ATCGATGAATTCGAGCTCG |
| Pol3degronf | TGACATTTTTTATATGCGGGTTAAGGTTAAAAAAGAGCTGCAGGAGAAAGTAGAACAATTAAGCAAATGG CGTACGCTGCAGGTCGAC |
| Pol3degronr | ATCCACCAACATGCAAAAAGTTGTTAGCCTTTCTTAATCCTAATATGATGTGCCACCCTATCGTTTTTTA ATCGATGAATTCGAGCTCG |
| Pol1tagf | GTGGACGTCGCTACGTTGATATGACTAGCATATTTGATTTCATGCTAAATCGGATCCCCGGGTTAATTAA |
| pol1tagr | CTATATAGAATATTCATGAGATCACACAACACATACAAAATACTTACCTAGAATTCGAGCTCGTTTAAAC |
| pol1WTF | GATTTTGTTGTAGATGACGATGGT |
| Pol12a2F | GATTTTGTTGTAGATGCCGATGGC |

**SI References**

1.	Gan, H., Serra-Cardona, A., Hua, X., Zhou, H., Labib, K., Yu, C. and Zhang, Z. (2018) The Mcm2-Ctf4-Polalpha Axis Facilitates Parental Histone H3-H4 Transfer to Lagging Strands. *Mol Cell*, **72**, 140-151 e143.

2.	Yu, C., Gan, H., Serra-Cardona, A., Zhang, L., Gan, S., Sharma, S., Johansson, E., Chabes, A., Xu, R.M. and Zhang, Z. (2018) A mechanism for preventing asymmetric histone segregation onto replicating DNA strands. *Science*, **361**, 1386-1389.

3.	Janke, R., King, G.A., Kupiec, M. and Rine, J. (2018) Pivotal roles of PCNA loading and unloading in heterochromatin function. *Proc Natl Acad Sci U S A*, **115**, E2030-E2039.

4.	Tian, C., Zhang, Q., Jia, J., Zhou, J., Zhang, Z., Karri, S., Jiang, J., Dickinson, Q., Yao, Y., Tang, X. *et al.* (2024) DNA polymerase delta governs parental histone transfer to DNA replication lagging strand. *Proc Natl Acad Sci U S A*, **121**, e2400610121.

5.	Huang, S., Zhou, H., Katzmann, D., Hochstrasser, M., Atanasova, E. and Zhang, Z. (2005) Rtt106p is a histone chaperone involved in heterochromatin-mediated silencing. *Proc Natl Acad Sci U S A*, **102**, 13410-13415.
